# The Potential Distribution of *Phytophthora nicotianae* in China on the Basis of MaxEnt Model Analysis

**DOI:** 10.1002/ece3.72487

**Published:** 2025-12-03

**Authors:** Chaolong Bi, Minggang Li, Hua Chen, Jiangyuan Zhao, Hongbo Zha, Dexi Wu, Fang Zhao, Peiwen Yang

**Affiliations:** ^1^ College of Plant Protection Yunnan Agricultural University Kunming China; ^2^ Yunnan Institute of Microbiology, Key Laboratory for Southwest Microbial Diversity of the Ministry of Education, School of Life Sciences Yunnan University Kunming China; ^3^ Zhaotong Tobacco Company of Yunnan Province Zhaotong China; ^4^ Lnstitute of Agricultural Environmental Resources Yunnan Academy of Agricultural Sciences Kunming China

**Keywords:** China, climate change, habitat suitability prediction, MaxEnt, *P. nicotianae*, XGBoost‐SHAP

## Abstract

Tobacco black shank, caused by *Phytophthora nicotianae*, is characterized by rapid outbreak onset and high control difficulty, posing a persistent threat to tobacco production in China. To assess its potential geographic distribution and management risks under climate change, this study employed 410 verified occurrence records and 32 environmental variables to construct a Maximum Entropy (MaxEnt) model, with key variable interpretation supported by an XGBoost–SHAP framework. The model simulated a suitable habitat under current conditions and four CMIP6 climate scenarios (SSP1‐2.6, SSP2‐4.5, SSP3‐7.0, and SSP5‐8.5) from 2021 to 2100. The optimized model (AUC = 0.959) identified eight key environmental predictors, including the minimum temperature of the coldest month (bio6), annual precipitation (bio12), temperature seasonality (bio4), precipitation of the wettest month (bio13), precipitation of the driest month (bio14), elevation, slope, and land use type. Response curves and SHAP dependence plots revealed clear ecological thresholds, such as a notable increase in suitability when bio6 exceeds 0°C, elevation ranges from 800 to 1500 m, and both bio13 and bio14 fall within specific precipitation intervals. Future projections showed an overall expansion of suitable areas, with the largest extent (165.54 × 10^4^ km^2^) under the SSP2‐4.5 scenario and a northeastward shift in habitat centroid. Overlaying predicted suitability with tobacco cultivation data revealed 28.35% spatial overlap, primarily in major growing regions such as Yunnan and Guizhou. These results clarify the critical role of temperature and moisture in shaping the pathogen's ecological niche and offer a quantitative foundation for risk‐based surveillance and region‐specific management of tobacco black shank.

## Graphical abstract

This study uses the MaxEnt model to predict the potential distribution of *Phytophthora nicotianae*, the pathogen responsible for tobacco black shank disease, across China under future climate scenarios. Our model identifies key environmental factors, including temperature, precipitation, and topography, that shape the pathogen's ecological niche. The predictions suggest an expansion of suitable habitats under climate change, with a shift in the disease's geographic distribution, and are combined with actual tobacco cultivation areas to perform a risk assessment, emphasizing the need for targeted disease management in major tobacco‐producing regions.

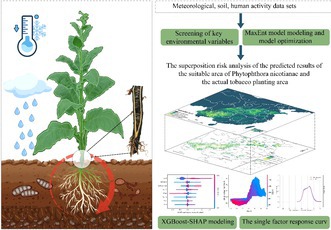

## 1 Introduction

Over the past 100 years, global temperatures have risen by nearly 1°C, with the rate of warming accelerating over the past 30 years. Climate change has had profound effects on species distribution patterns and ecosystem functions, altering ecosystem structures, biodiversity, and the genetic diversity of populations. It has become one of the key issues in global ecological research (Jump and Peñuelas 2005; Pio et al. 2014; Zhao et al. 2021). In particular, plant pathogens such as *P. nicotianae* , which causes tobacco black shank disease, are increasingly influenced by shifting climate patterns, making it critical to understand how climate change may alter their distribution. *P. nicotianae* , an oomycete plant pathogen in the genus *Phytophthora*, is widely distributed across the globe, particularly in tobacco‐producing regions of eastern, southern, and southwestern China, where it is highly prevalent (Judelson and Blanco 2005). However, the precise distribution of this pathogen remains unclear. Studies have shown that *P. nicotianae* is highly adaptable, preferring warm and humid environments, and its lifecycle is closely linked to soil moisture conditions. Under high moisture conditions, it produces motile zoospores, which rapidly spread and invade tobacco plants through the roots, causing black shank disease characterized by root rot and stem base decay (Demirjian et al. 2022). Under favorable environmental conditions, the pathogen can spread rapidly in cultivation areas, resulting in tobacco black shank disease. This disease presents rapid outbreaks and has a very short control window, posing significant challenges for effective disease management. Infected tobacco plants exhibit root rot, wilting, and even death, significantly reducing both yield and quality, causing severe economic losses and threatening the healthy development of tobacco farming in China. The concentration of the pathogen in the soil is considered one of the key factors determining disease incidence, with higher concentrations positively correlating with the prevalence of the disease (Batista et al. 2024).

Despite its severe impact, the geographic distribution of *P. nicotianae* in China remains insufficiently understood. Previous studies have mainly focused on pathogen biology, molecular mechanisms, and chemical or genetic control, whereas systematic research on its ecological niche and potential spread under climate change is still limited. This knowledge gap restricts the ability to anticipate outbreaks and design targeted management strategies. Given the increasing vulnerability of tobacco production to climate variability and the critical role of temperature and soil moisture in driving pathogen dynamics, it is essential to predict the future distribution of *P. nicotianae* to inform effective management strategies.

To bridge this gap, this study employs species distribution modeling (SDM) to predict the potential distribution of *Phytophthora nicotianae* in China. By integrating occurrence records, environmental variables, and climate change scenarios, the study aims to identify key environmental factors that influence the pathogen's distribution. Additionally, the research will define the value ranges of these factors associated with suitable habitats. This approach not only forecasts the spread of *P. nicotianae* but also lays a scientific foundation for risk assessments and region‐specific management strategies.

Species distribution models (SDMs), also known as ecological niche models (ENMs), are mathematical tools that predict species' geographic distributions on the basis of occurrence data and environmental variables (Elith and Leathwick 2009). These models, developed by Phillips et al. using the principle of maximum entropy, estimate the ecological environment required by the species and project suitable habitats across different spatial and temporal scales (Li and Qin 2018; Phillips et al. 2006). The model estimates the ecological environment required by the species and projects the results across different spatiotemporal scales to predict current or future species distributions. Typically, the model results reflect the species' suitable habitat distribution across large spatial scales (Zhu et al. 2013). As a vital tool for studying the effects of environmental variables on species distribution, SDMs are widely used to predict the potential distribution areas of invasive species, assess the impact of climate change on species abundance, and evaluate habitat suitability (Peterson 2011). Commonly used SDMs include the Genetic Algorithm for Rule Set Prediction (GARP), Maximum Entropy (MaxEnt), Bioclimatic Prediction (BioClim), Generalized Linear Models (GLM), Random Forest (RF), and Ecological Niche Factor Analysis (ENFA) (Gu et al. 2024). Among these, the MaxEnt model, a widely applied SDM, simulates species' potential distributions by combining species occurrence points and environmental variables with machine learning and the maximum entropy principle (Phillips et al. 2004). This model requires only species occurrence data and environmental background data, effectively handling complex interactions between variables (Kong et al. 2019). MaxEnt is highly tolerant of small sample sizes, irregular sampling, and biased data, and can provide excellent predictive performance even with limited species distribution data (Fourcade et al. 2014; Hernandez et al. 2006; Urbani et al. 2015). Therefore, MaxEnt has been widely used in various fields, including potential plant cultivation zones (Del Río et al. 2021; Liu et al. 2022), animal and plant habitats (Lu et al. 2021; Zhou et al. 2022), and plant pathogen predictions (Bo et al. 2024; Tao et al. 2021; Wang et al. 2020).

In this study, we used the MaxEnt model to predict the suitable habitat for tobacco black shank disease in China. This study quantitatively describes the suitable environmental conditions for *P. nicotianae* , analyzes climate data under different environmental scenarios, and forecasts future range shifts in suitable habitats. The objective is to bridge the gap in understanding the spatial and temporal dynamics of the disease, thereby informing disease management and control. By combining the actual spatial pattern of tobacco cultivation, we assess the spatiotemporal risk of the disease. Through analyzing the driving effects of environmental factors on pathogen suitability, this study provides a theoretical basis for precision management, monitoring, early warning, and ecological control, contributing to mitigating the ecological and economic pressures on tobacco production systems under climate change. Ultimately, the goal of this study is to identify the most critical environmental drivers of pathogen suitability, providing actionable insights for region‐specific management strategies, early warnings, and ecological control measures. This will provide a theoretical foundation for disease prediction, forecasting, and effective control strategies.

## 2 Materials and Methods

### 2.1 Data Sources and Distribution Point Processing of Termitomyces

**FIGURE 1:**
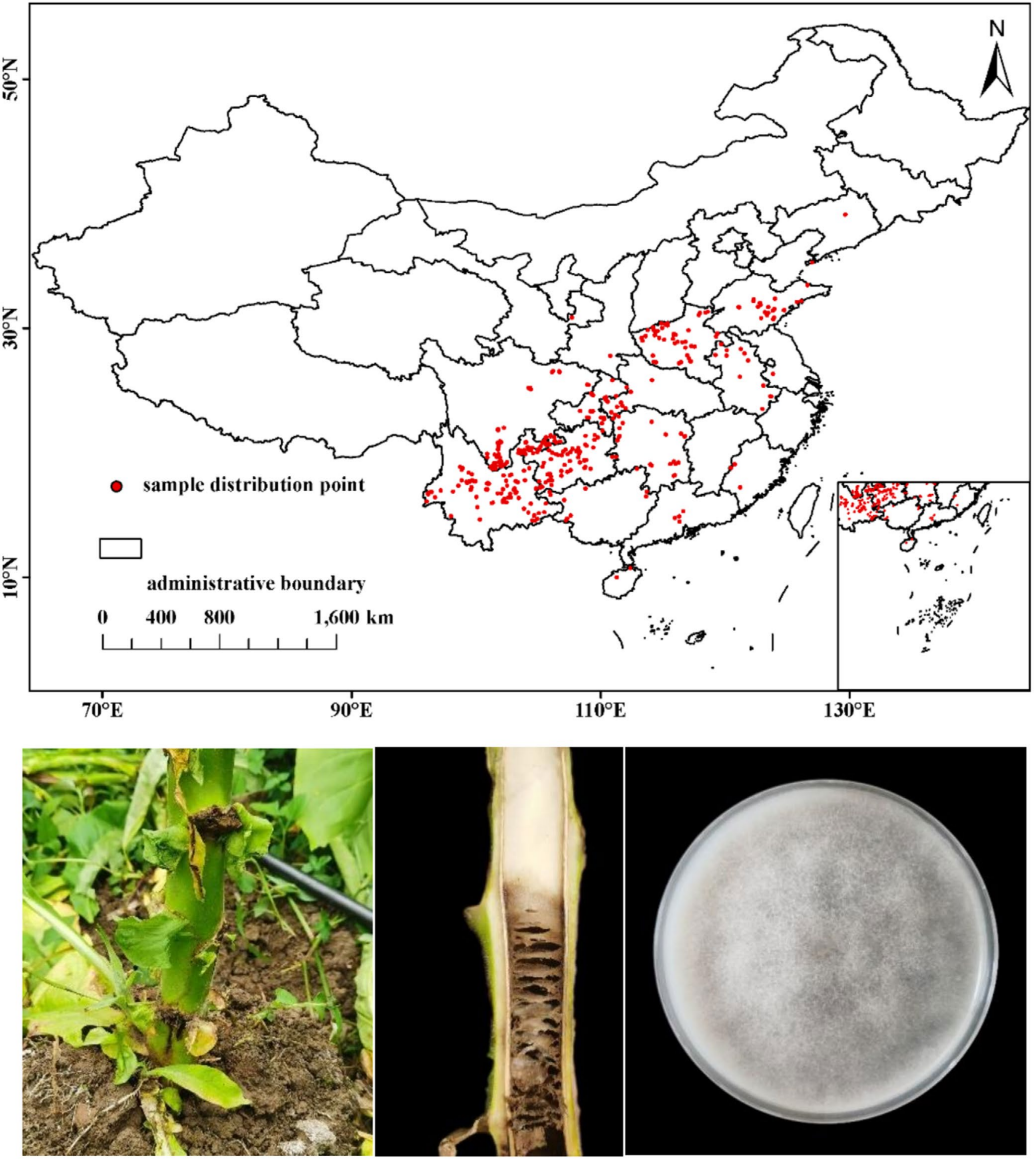
The distribution points of *P. nicotianae* in China and the incidence of tobacco black shank and the colony morphology of pathogenic bacteria.

To assess the impact of climate and environmental factors on the geographic distribution of *P. nicotianae* in different regions of China, the study collected climate and soil data. The species distribution data were sourced as follows: (1) In June–September 2024, the research team conducted a survey and identification of *P. nicotianae* in Zhaotong City, Yunnan Province. In each major tobacco‐growing county, five towns were surveyed, and three villages were surveyed per town, ultimately identifying 116 distribution points for *P. nicotianae* ; (2) Relevant data were obtained from the Global Biodiversity Information Facility (GBIF, https://www.gbif.org) species distribution database; (3) Relevant literature on tobacco black shank disease and *P. nicotianae* was searched using keywords. Indoor and greenhouse experimental data were excluded, and only distribution data for *P. nicotianae* in natural environments were retained. For literature where geographic coordinates were not explicitly provided, PyCharm was used to call the Gaode Open Platform Geocoding API to obtain coordinates from the collection sites listed in the Excel file. For sites without coordinates, the POI Search API was used to find coordinates of tobacco leaf stations within a 10 km radius. The distribution points were then cross‐checked for accuracy. To avoid model overfitting caused by clustering of disease distribution points, the initially obtained 531 distribution points were input into the ENMtools package, and duplicate or invalid data were removed (Tang et al. 2023). A buffer radius of 5.0 km was set in ArcGIS to improve the spatial uniformity of the distribution points, preventing environmental bias and overfitting caused by spatial autocorrelation of distribution data. After filtering, 410 valid distribution points for *P. nicotianae* were retained and saved in CSV format for subsequent modeling analysis (Figure 1). For map creation, the study used the national administrative boundary data from the National Geospatial Information Center of China, with map approval number: GS(2024)0650, as the base map for the study.

### 2.2 Screening of Environmental Variables

In order to accurately predict the potential distribution of *P. nicotianae* , it is essential to choose environmental variables that are closely linked to the pathogen's survival, transmission, and ecological niche. These variables directly influence the pathogen's lifecycle and infectivity. Climatic factors such as temperature and humidity are widely recognized as central determinants of the pathogen's survival and spread. Furthermore, variables such as elevation and slope are important for identifying favorable microclimates, which directly influence the pathogen's distribution and growth. Land use type reflects the impact of human activity on the pathogen's spread, and as such, these variables were incorporated into the model to ensure reliable predictions of the pathogen's distribution and risk under varying climate scenarios. The sources and methods for variable processing are outlined below.

Historical climate data (1970–2000) comprising 19 bioclimatic variables (Appendix Table 1 in the Supporting Information) were sourced from WorldClim 2.1 (https://worldclim.org/). For future projections, the Beijing Climate Center‐Climate System Model 2‐Medium Resolution (BCC‐CSM2‐MR) model—part of the Coupled Model Intercomparison Project Phase 6 (CMIP6) and an improvement over the Coupled Model Intercomparison Project Phase 5 (CMIP5)'s Beijing Climate Center‐Climate System Model 1.1m (BCC‐CSM1.1m) in simulating temperature and precipitation—was used across four climate change scenarios from the Shared Socioeconomic Pathways (SSPs) (SSP1‐2.6, SSP2‐4.5, SSP3‐7.0, SSP5‐8.5), representing pathways from sustainability to high fossil fuel dependency (Ostad‐Ali‐Askari et al. 2020; Shi et al. 2023; Su et al. 2021; Wu et al. 2019). Predictions were made for 2021–2100, divided into four 20‐year intervals.

Soil organic matter data were obtained from the dataset shared by Dai Yongyong and Shangguan Wei via the National Tibetan Plateau Science Data Center (Shangguan et al. 2013) (see Appendix Table 2 in the Supporting Information). To reduce multicollinearity, variables with contribution > 1 and correlation < 0.8 were retained using MaxEnt‐based filtering.

Given the disease's prevalence in low‐lying areas (Hidayah et al. 2021) elevation, slope, and aspect were derived from GEBCO's 2024 DEM (https://www.gebco.net/data‐products/gridded‐bathymetry‐data) using ArcGIS (Giuseppi et al. 2023). Land use data from 1990 to 2022, provided by Yang Jie and Huang Xin (Wuhan University), were derived from 335,709 Landsat images using Google Earth Engine, producing the CLDC dataset with nine land cover types (Jie and Xin 2023).

To assess the practical impact of *P. nicotianae* on the tobacco industry, actual tobacco cultivation data from EarthStat (http://www.earthstat.org/) were integrated. Overlay analysis of predicted suitability and cultivation zones helps identify high‐risk areas and provides spatial guidance for disease warning, planting optimization, and climate‐resilient agricultural planning.

### 2.3 Screening of Environmental Variables

Prior to modeling, all processed raster layers were converted to ASC format using the “Raster to ASCII” tool in SDM Toolbox (Brown et al. 2017; Li et al. 2024). Species occurrence points were spatially filtered with ENMTools to eliminate spatial autocorrelation, thereby reducing the risk of model overfitting. A total of 410 spatially independent points were retained for subsequent modeling (Warren et al. 2010).

To improve prediction accuracy and model robustness, 32 candidate environmental variables were evaluated on the basis of three criteria: contribution rate, information gain, and multicollinearity. Variables contributing less than 1% were removed on the basis of the MaxEnt output. Jackknife analysis was further used to exclude variables with low training and testing gains, ensuring strong interpretability and generalizability. Pearson correlation coefficients were computed using ENMTools, and among pairs with |*r*| ≥ 0.8, only those with higher contribution and greater information gain were retained (Phillips et al. 2017). Multicollinearity was also assessed using the vifstep() function from the usdm package in R, with a threshold of VIF > 10; 17 variables with VIF < 10 were ultimately selected. To evaluate nonlinear associations and reduce redundancy, 5000 non‐missing samples were randomly extracted from the raster stack using the raster package, and Spearman rank correlations were calculated (Pradhan 2016).

MaxEnt was selected for its superior ecological realism and predictive consistency compared to other models (e.g., GLM, GBM, CTA, ANN, SRE, FDA, and RF) (Zhao et al. 2021). Model optimization was conducted using ENMeval 2.0.4 to reduce overfitting and improve simplicity. A total of 36 model combinations were tested, derived from four feature classes (FC) types (L, LQ, LQH, LQHP) and nine regularization multipliers (RM: 0.5–4.0 at 0.5 intervals) (Muscarella et al. 2014; Radosavljevic and Anderson 2014). FC determines the complexity of response curves, whereas RM penalizes complexity to balance generalization and fit.

Model performance was evaluated using 10 bootstrap replicates, with species occurrence points randomly split into 75% for training and 25% for validation. The area under the ROC curve (AUC) was used as the evaluation metric: 0.50–0.60 (fail), 0.60–0.70 (poor), 0.70–0.80 (good), 0.80–0.90 (very good), and 0.90–1.00 (excellent).

### 2.4 Grading of Suitable Areas of *P. nicotianae*

The ASC‐format output from the MaxEnt model was imported into a GIS platform, converted into raster format, and overlaid with administrative boundaries and topographic maps for spatial visualization. Habitat suitability was reclassified using the GIS reclassification tool on the basis of habitat suitability index (HSI, or *p*‐value), with four thresholds: high suitability (*p* ≥ 0.6), moderate (0.4 ≤ *p* < 0.6), low (0.2 ≤ *p* < 0.4), and unsuitable (*p* < 0.2) (Shi et al. 2023). Grid counts in each category were used to calculate suitable habitat areas under both current and future climate scenarios (SSP1‐2.6, SSP2‐4.5, SSP3‐7.0, SSP5‐8.5).

To improve the model's practical value in targeted disease management and early warning applications, spatial distribution data of major tobacco cultivation regions in China were incorporated. A Tobacco Black Shank Disease Risk Index (TBSRI) was developed by multiplying the MaxEnt‐derived HSI values with the corresponding rasterized tobacco planting area data, allowing quantification of spatial disease risk. Regions with higher TBSRI values indicate a convergence of favorable pathogen habitat and intensive tobacco cultivation, representing priority areas for surveillance, intervention, and resource allocation.

### 2.5 XGBoost‐SHAP Modeling and Variable Interpretation

To enhance the predictive accuracy and interpretability of species distribution modeling, this study developed an XGBoost–SHAP interpretation framework in Python, integrating two widely used libraries: xgboost and shap. These tools have been increasingly adopted in ecosystem service assessments and species suitability modeling because of their strong nonlinear fitting capacity and high explanatory power (Schoonemann et al. 2024; Zhou et al. 2024).

SHAP analysis was applied to quantify the marginal effect and directional influence (positive or negative) of each variable on habitat suitability, as well as to identify potential interactions among variables. This approach not only provides average contribution values for each predictor but also reveals variable interactions that influence species distribution patterns. By overcoming the limitations of traditional regression models on the basis of linear assumptions, the XGBoost–SHAP framework offers a transparent and ecologically robust method for interpreting complex environmental drivers in species distribution modeling.

In this framework, the species suitability probability map generated by MaxEnt served as the response variable. Raster data for all environmental predictors across the study area were extracted, standardized for spatial resolution, and uniformly read using the rasterio library. All ASC‐format data were flattened into a feature matrix after handling missing values to ensure training data quality. An XGBoost regression model was then fitted to capture the complex nonlinear relationships between environmental variables and species suitability.

## 3 Results

### 3.1 Simulation Accuracy Test and Key Environmental Variables Screening

To improve prediction accuracy and model robustness, 55 candidate environmental variables were evaluated on the basis of three criteria, including 19 climate variables (Appendix Table 1 in the Supporting Information), 32 soil environmental variables (Appendix Table 2 in the Supporting Information), as well as elevation, slope, aspect, and land use data. These variables were preliminarily screened and reduced to 32 variables through contributions, information gain, and multicollinearity. Further screening results showed that among them, bio6 (minimum temperature of the coldest month) showed the highest contribution (27.6%), followed by bio14 (precipitation of the driest month, 20.9%) and land use type (12.8%), with elevation and bio12 (annual precipitation) also playing notable roles. Environmental factors with contribution values greater than 1 are listed in Table 1. Jackknife analysis (Appendix Figure 1 in the Supporting Information) identified land use as the most irreplaceable predictor, whereas bio6, bio11, bio12, and bio1 were the top‐performing climatic variables, underscoring the limiting effects of extreme low temperatures and annual variability. Soil attributes such as cation exchange capacity and elevation contributed moderately through interactions, whereas factors like pH, gravel content, and porosity had minimal direct impact. Correlation and multicollinearity analysis (Appendix Figure 2 in the Supporting Information, Appendix Table 3 in the Supporting Information) revealed strong collinearity among several climatic variables—particularly bio1, bio5, bio6, and bio10—but weak correlations among soil variables, except for fraction and hectares, which showed moderately elevated VIF values. On the basis of a combined assessment of variable contribution, Jackknife gain, Pearson correlation, and VIF thresholds, eight key predictors were selected for MaxEnt modeling: bio2, bio4, bio6, bio13, bio14, elevation, slope, and land use type.

**TABLE 1:** Contribution screening results of environmental factors.

Variable	Percent contribution/%	Permutation importance/%	Variable	Percent contribution/%	Permutation importance/%
bio 6	27.6	0.7	cw	0.6	0.8
bio 14	21.4	7.8	bio 11	0.4	3.8
Land use type	12.8	3.1	grav	0.4	0.3
Elevation	8.8	11.5	bio 15	0.3	1.6
bio 12	8.7	2.7	pH	0.3	0.7
Slope	4.8	2.7	por	0.3	0.6
bio 19	2.3	13.2	bio 7	0.2	2.6
bio 13	2.2	11.1	bio 18	0.2	4.6
bio 4	1.7	1.2	som	0.2	0.5
bio 2	1.6	2.1	wc	0.2	0.6
bio 17	1	10.7	bio 9	0.1	2.9
Aspect	0.9	0.7	bio 10	0.1	0.7
bio 1	0.8	6.9	cec	0.1	0.3
bio 3	0.6	0.7	bio 16	0	0.1
bio 5	0.6	0.8	cw	0.6	0.8
bio 8	0.6	3.8	bio 11	0.4	3.8

*Note:* See Appendix Tables 1 and 2 in the Supporting Information for full abbreviations in the table.

### 3.2 Environmental Response Characteristics of Key Variables

The optimization results (Appendix Figure 3 in the Supporting Information) show that the feature combinations L and LQ have significantly lower Training AUC values compared to LQH and LQHP across all regularization multiplier (RM) values, indicating weaker discriminative ability. These combinations also exhibit lower Regularized Training Gain and higher Entropy, further suggesting poor fitting ability and the risk of overfitting. Additionally, they have larger Prevalence ranges and more ambiguous boundaries, leading to their exclusion from the final optimization process. Among the remaining combinations, LQH performed best at RM = 2.5, maintaining a good balance across multiple performance metrics, including AUC, Gain, Entropy, and Prevalence, while effectively controlling model complexity and avoiding overfitting. In contrast, the model using the default parameters (FC = LQHP, RM = 1) showed a decline in Training AUC and Regularized Training Gain, indicating a tendency for overfitting. After considering the model's discriminative ability, generalization, and stability, RM = 2.5 and FC = LQH were ultimately selected as the optimal parameter combination. This combination provides more accurate predictions and exhibits strong operational feasibility and reliability, making it suitable for future applications.

### 3.3 Environmental Response Characteristics of Key Variables

**FIGURE 2:**
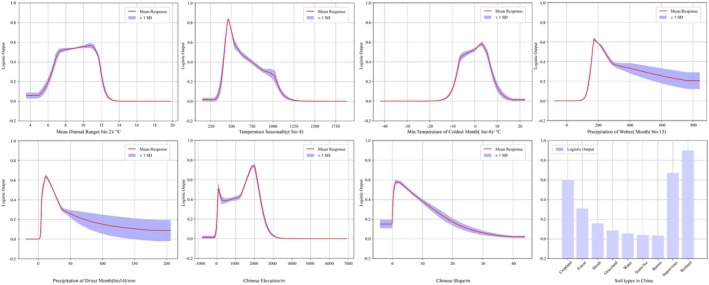
Response curves of eight environmental variables in the ecological niche model for *P. nicotianae* .

The ecological responses of eight key environmental variables—bio2, bio4, bio6, bio13, bio14, elevation, slope, and land use type—were analyzed using MaxEnt response curves and XGBoost–SHAP analysis (Figure 2, Appendix Figures 4 and 5 in the Supporting Information). Both methods showed consistent, nonlinear effects with clear threshold responses. Bio6 (minimum temperature of the coldest month) had the strongest impact, with species suitability increasing sharply above −6°C and peaking around 0°C, indicating a critical thermal threshold for overwintering survival. Bio13 and bio14 (precipitation in the wettest and driest months) exhibited optimal ranges (bio13: 150–270 mm, bio14: 5–30 mm) for suitable habitat, with negative effects observed under extreme wet or dry conditions.

In terms of elevation, both models identified mid‐elevations (1000–2000 m) as the most suitable for *P. nicotianae* , supporting the “mid‐domain optimum” hypothesis. Slope displayed strong nonlinear effects, with suitability peaking at low slopes (0°–2°), but varying on the basis of interactions with other variables. For land use, wetlands, farmlands, and impervious surfaces were the most suitable habitats (HSI > 0.6 in MaxEnt), with SHAP values indicating these land types provide favorable microenvironments for the species. Bio2 (mean diurnal range) and bio4 (temperature seasonality) showed negative correlations with suitability at higher values, suggesting the species prefers more climate‐stable regions.

The SHAP dependence plots (Appendix Figure 5 in the Supporting Information) further confirmed these findings by visualizing the marginal effects of key variables and revealing local ecological thresholds and interactions. These results provide valuable insights into the non‐linear relationships between environmental factors and species distribution, offering improved ecological interpretability and enhancing the accuracy of species niche modeling compared to traditional regression methods.

The MaxEnt model, constructed using eight ecological factors and optimized parameters, was run 10 times to evaluate performance. The results (Appendix Figure 6 in the Supporting Information) show that under historical climate conditions (1970–2020), the average AUC reached 0.959. These results indicate that the model performs well and is suitable for simulating potential habitats of *P. nicotianae* under current and future climate scenarios.

### 3.4 Environmental Response Characteristics of Key Variables

**FIGURE 3:**
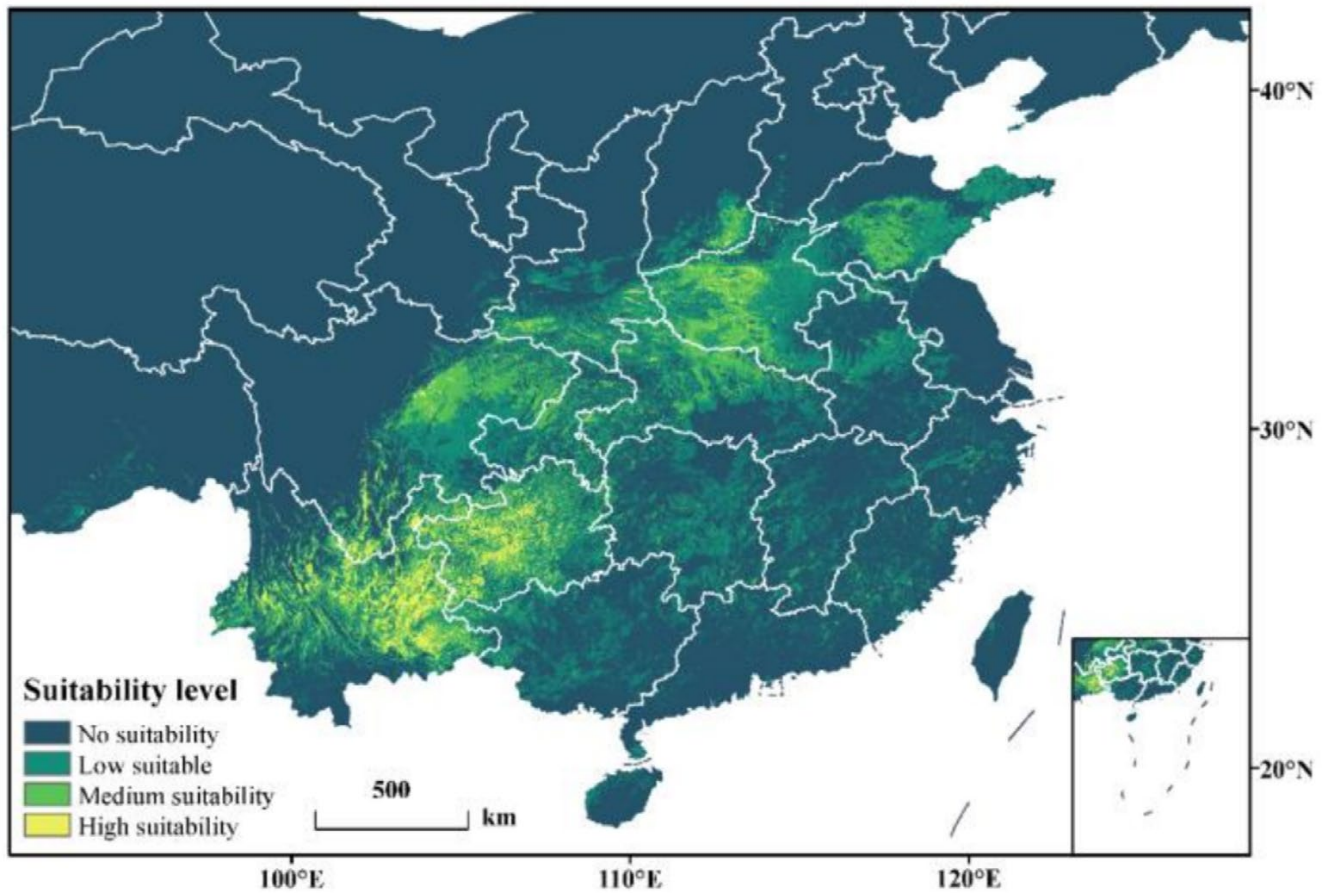
Potential geographical distribution of *P. nicotianae* under current climatic conditions.

The suitability area calculation results from the MaxEnt model (Figure 3) show that under current climate conditions, *P. nicotianae* is primarily distributed in the southern and eastern regions of China. The total suitable habitat area is 119.72 × 10^4^ km^2^, accounting for 12.47% of China's total land area. Among these, the Low suitable area is the largest, covering 81.51 × 10^4^ km^2^, and is distributed in most regions of Yunnan, Gui‐zhou, Chongqing, Hubei, Hunan, Henan, Guangxi, Jiangxi, Fujian, and Zhejiang. There are also smaller distributions in eastern Sichuan, parts of Shaanxi, southern Shanxi, southern Shandong, and northern Guangdong. Additionally, there are small distributions in Tibet, Hainan, Taiwan, Gansu, and Jiangsu. The Medium suitability area covers 28.69 × 10^4^ km^2^, whereas the High suitability area covers 9.52 × 10^4^ km^2^. The distribution of these two areas is quite similar, with the main distributions occurring in eastern Yunnan, Guizhou, eastern Sichuan, southern Henan, southern Shandong, southern Shanxi, southern Shaanxi, and northern Hubei.

### 3.5 Future Suitable Area Distribution and Centroid Migration

**FIGURE 4:**
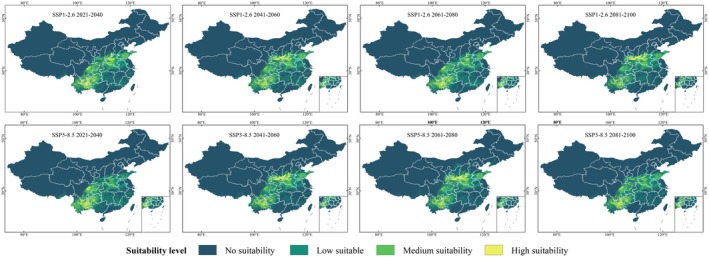
Predicted distribution of *P. nicotianae* in China.

As shown in Figure 4 and Appendix Figure 7 in the Supporting Information, the overall distribution of suitable habitats for *P. nicotianae* remains relatively stable, concentrated primarily in eastern and southern China across all four emission scenarios. Under SSP1‐2.6, medium and high suitability areas expand notably in Henan, Shaanxi, Shandong, and Anhui, as well as in Yunnan, Guizhou, and Sichuan. A northeastward shift is observed, with suitability decreasing in Yunnan and Sichuan but increasing in Henan, Shanxi, and Shaanxi over time. Under SSP2‐4.5, high suitability regions shift from southwestern provinces (Yunnan, Guizhou, Sichuan) to central‐northern regions (Henan, Shanxi, Shaanxi) in most periods. The SSP3‐7.0 scenario shows the most substantial expansion in the Sichuan Basin around 2041–2060. Under SSP5‐8.5, the total suitable area is smaller than in other scenarios but still exceeds the current baseline. Yunnan and Guizhou maintain stable suitability, whereas Sichuan and central‐northern provinces exhibit more dynamic temporal fluctuations.

As shown in Table 2, under the SSP1‐2.6 scenario, the suitable habitat area for *P. nicotianae* reaches 166.69 × 10^4^ km^2^ (17.36% of China's land area) between 2021 and 2040, an increase of 46.97 × 10^4^ km^2^ compared to the current period. The peak occurs between 2041 and 2060 at 168.83 × 10^4^ km^2^, before contracting to 145.39 × 10^4^ km^2^ by 2081–2100. The SSP2‐4.5 scenario shows the largest increase in suitable habitat (181.02 × 10^4^ km^2^) between 2081 and 2100, accounting for 18.85% of China's land area. This scenario also has the largest high suitability area (18.7 × 10^4^ km^2^). Overall, the suitable habitat under SSP2‐4.5 gradually expands, with average suitability occupying 16.65% of China's land area. In contrast, the SSP5‐8.5 scenario shows fluctuating trends, with high and medium suitability areas peaking between 2061 and 2080 at 16.26 × 10^4^ and 43.56 × 10^4^ km^2^, respectively. Across all four carbon emission scenarios, the suitable habitat area increases in all suitability categories, suggesting a rise in tobacco black shank disease risk in China. The SSP2‐4.5 scenario has the largest average suitable habitat area (165.54 × 10^4^ km^2^), whereas SSP5‐8.5 has the smallest (154.68 × 10^4^ km^2^), but with more fluctuations.

**TABLE 2:** Statistics of the suitable area of *P. nicotianae* in China under different climate scenarios in different periods.

Climate change scenario	Area (×10^4^ km^2^)						Proportion %
	No suitability	Low suitability	Medium suitability	High suitability	Net change	Total suitable area	
Current condition	840.6	81.51	28.69	9.52	NA	119.72	12.47
SSP1‐2.62021–2040	793.63	100.81	49.43	16.45	46.97	166.69	17.36
SSP1‐2.62041–2060	791.50	104.17	46.78	17.88	49.10	168.83	17.58
SSP1‐2.62061–2080	801.16	102.29	43.80	13.06	39.44	159.15	16.57
SSP1‐2.62081–2100	814.92	94.48	36.89	14.02	25.68	145.39	15.14
SSP2‐4.52021–2040	801.77	93.76	47.35	17.44	38.83	158.55	16.51
SSP2‐4.52041–2060	800.5	99.01	44.27	16.54	40.10	159.82	16.64
SSP2‐4.52061–2080	797.55	104.86	44.73	13.18	43.05	162.77	16.95
SSP2‐4.52081–2100	779.29	113.49	48.83	18.70	61.31	181.02	18.85
SSP3‐7.02021–2040	794.79	100.73	47.21	17.60	45.81	165.54	17.24
SSP3‐7.02041–2060	809.41	98.19	38.96	13.76	31.19	150.91	15.71
SSP3‐7.02061–2080	805.1	100.83	41.35	13.04	35.50	155.22	16.16
SSP3‐7.02081–2100	792.55	108.67	44.00	15.10	48.05	167.77	17.47
SSP5‐8.52021–2040	803.58	102.46	41.41	12.87	37.02	156.74	16.32
SSP5‐8.52041–2060	806.6	95.88	41.86	15.97	34.00	153.71	16.01
SSP5‐8.52061–2080	800.24	100.27	43.56	16.26	40.36	160.09	16.67
SSP5‐8.52081–2100	812.14	95.34	41.39	11.45	28.46	148.18	15.43

**FIGURE 5:**
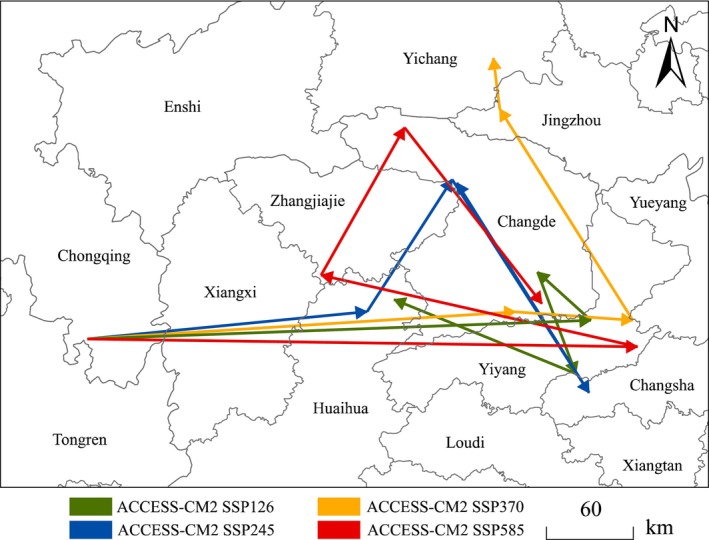
Migration of the center of suitable habitat for *P. nicotianae* in China.

As shown in Figure 5, under current conditions, the centroid of the suitable habitat for *P. nicotianae* in China is located in Chongqing (N 108.736775, E 28.508998), with its overall movement primarily toward the southern regions of China. In the future, under the four carbon emission scenarios, the centroid shifts eastward in the 2021–2040 period. Among the scenarios, the SSP5‐8.5 scenario shows the greatest shift, moving 383.22 km, followed by the SSP1‐2.6 scenario, which moves 350.89 km. Between 2041 and 2060, the centroid shifts northeastward under the SSP2‐4.5 scenario, whereas it continues to shift eastward under the SSP2‐4.5 scenario. In contrast, the centroids of the remaining two scenarios move northwestward. Ultimately, in the 2081–2100 period, the centroid in the SSP1‐2.6 scenario is located in Huaihua, whereas the centroid in the SSP2‐4.5 scenario moves from Changde to Changsha in the 2061–2080 period, but returns to Changde in the 2081–2100 period. The centroid of the SSP3‐7.0 scenario moves eastward and then northward, remaining in Yichang between 2081 and 2100. Similarly, the centroid of the SSP5‐8.5 scenario also remains in Changde by the end of the century.

**FIGURE 6:**
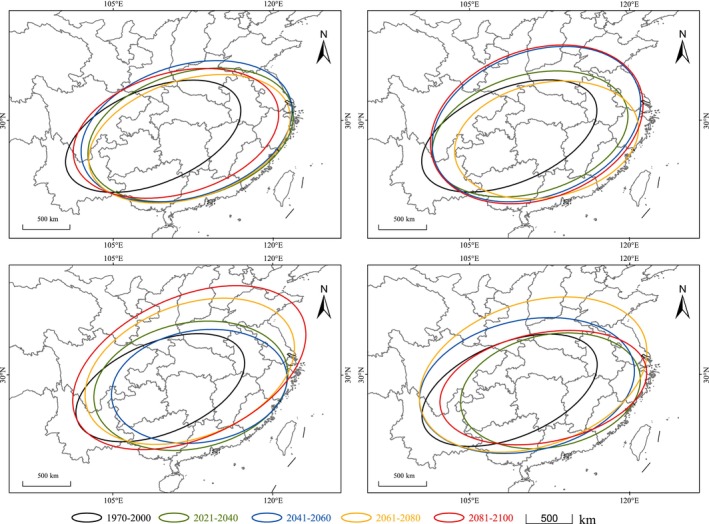
Scope of the suitable area of *P. nicotianae* in the future. (a) The change of suitable habitat range of *P. nicotianae* in China under the ACCESS‐CM2 SSP1‐2.6 scenario. (b) The change of suitable habitat range of *P. nicotianae* in China under the ACCESS‐CM2 SSP2‐4.5 scenario. (c) The change of suitable habitat range of *P. nicotianae* in China under the ACCESS‐CM2 SSP3‐7.0 scenario. (d) The change of suitable habitat range of *P. nicotianae* in China under the ACCESS‐CM2 SSP5‐8.5 scenario.

Under the SSP1‐2.6 climate scenario, the future range of *P. nicotianae* 's suitable habitat shows an overall trend of expansion followed by contraction (Figure 6a). Among the four future climate scenarios, the overall change is the most stable, but compared to the current climate scenario, the suitable habitat range of *P. nicotianae* expands under the SSP1‐2.6 scenario, with a general trend of eastward expansion in China. Under the SSP2‐4.5 climate scenario (Figure 6b), the suitable habitat ranges in the periods 2041–2060 and 2081–2100 show significant overlap. In the SSP3‐7.0 climate scenario, the changes in the suitable habitat area for *P. nicotianae* are relatively large. Except for a slight contraction in the suitable habitat area between 2041 and 2100 compared to 2021–2040, the suitable habitat area for *P. nicotianae* increases in the remaining periods compared to the previous period (Figure 6c). Among all the climate scenarios, the SSP5‐8.5 scenario shows an expansion of *P. nicotianae* 's suitable habitat over three consecutive periods, followed by a contraction in the 2081–2100 period (Figure 6d). Therefore, during the 2061–2080 period, the suitable habitat area for *P. nicotianae* reaches its peak under this scenario.

### 3.6 Future Suitable Area Distribution and Centroid Migration

**FIGURE 7:**
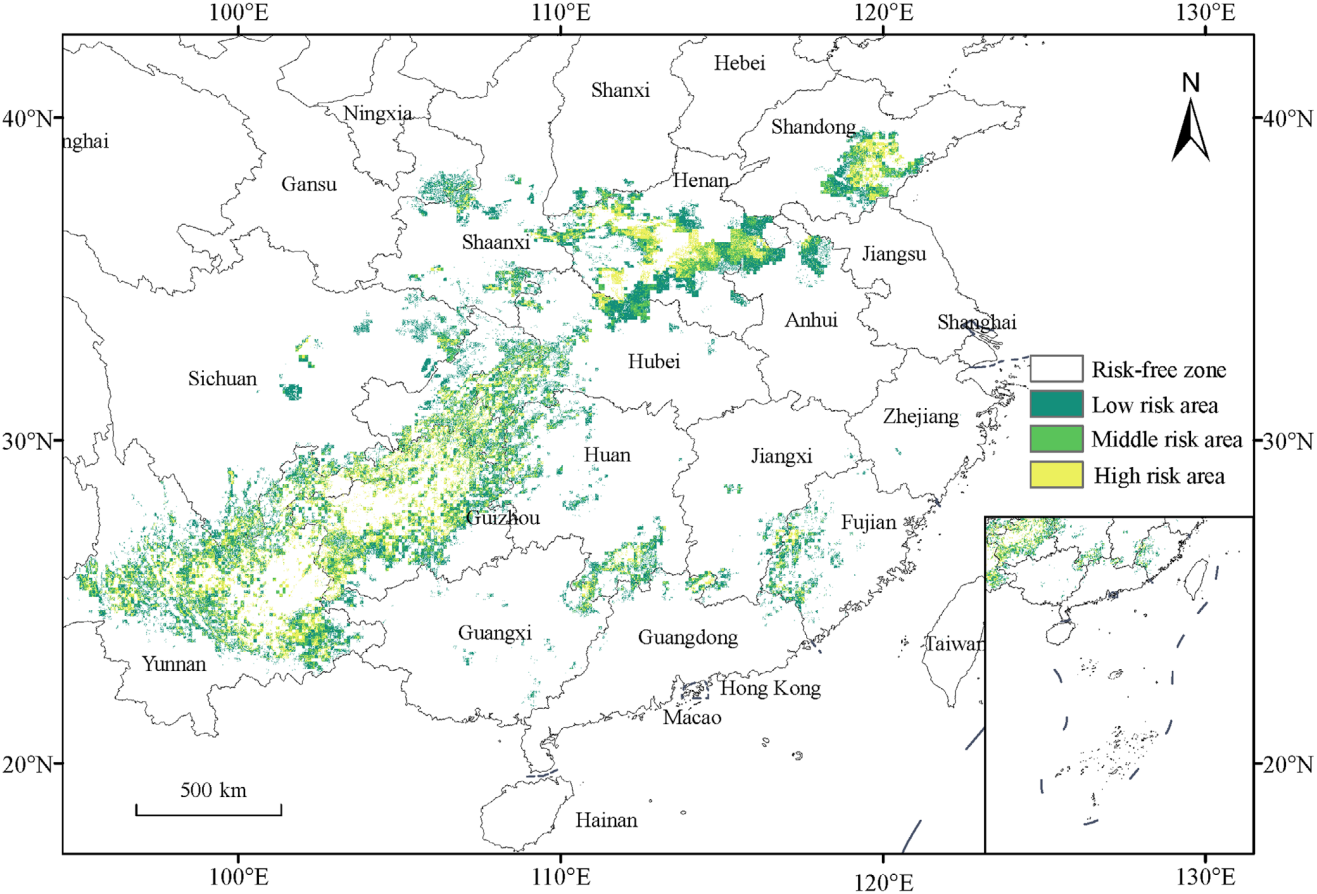
Risk prediction of tobacco black shank disease.

In real‐world production, the tobacco cultivation areas, as hosts for *P. nicotianae* , have a significant impact on the distribution of the pathogen. To clarify the distribution of *P. nicotianae* in tobacco cultivation areas in actual production settings, this study incorporated the actual tobacco planting area to construct a MaxEnt model. The model demonstrated an AUC value of 0.966. The results of the tobacco black shank disease risk overlay analysis are shown in Figure 7. Although *P. nicotianae* 's suitable habitat is widely distributed across the eastern and southern regions of China, the areas that require focused monitoring in actual production are relatively few, covering 33.94 × 10^4^ km^2^, which accounts for 28.35% of the total suitable habitat area for *P. nicotianae* and 3.53% of China's total land area. The high‐risk areas for tobacco black shank disease currently observed in China are distributed in a band from Yunnan to Shandong. The regions with higher risk levels include eastern Yunnan, central, eastern, and northern Guizhou, southeastern Chongqing, central Henan, and eastern Shandong, with sporadic high‐risk areas in parts of Hunan, Fujian, Shanxi, and Gansu. The low‐risk area spans 16.05 × 10^4^ km^2^, whereas the medium‐risk area covers 8.36 × 10^4^ km^2^, and the high‐risk area measures 9.52 × 10^4^ km^2^. Among these regions, Yunnan and Guizhou have the largest high‐risk areas, though they are relatively scattered. In contrast, Henan and Shandong have more concentrated high‐risk areas, making them key regions for disease prevention and control.

## 4 Discussion

We found that research on *P. nicotianae* rarely focuses on its geographic distribution, with most studies only providing location data down to the township level. Consequently, there are relatively few publications with precise coordinates, and usable point data are limited. For the relatively limited research in this field. To address this issue, we creatively used PyCharm to call the POI Search API and query the coordinates of tobacco leaf stations within a 10 km radius. In China, farmers typically choose to plant tobacco near tobacco leaf stations to facilitate harvesting or to receive technical support from professionals. Therefore, using the coordinates of tobacco leaf stations within administrative regions in the literature is the best approach to determine the distribution points of *P. nicotianae* . This innovative method solved the problem of insufficient geographic distribution data for the pathogen, enabling the successful application of the MaxEnt model. After incorporating these coordinates into the model, it performed well. This approach can be extended to predict other tobacco pathogens and may also be applied to other species with limited precise coordinate data in the existing literature.

The Area Under the ROC Curve (AUC) is a key evaluation metric, particularly for binary classification problems. AUC measures the model's ability to rank samples, specifically its ability to prioritize positive samples over negative ones. Despite high AUC values (> 0.94 for training and testing), MaxEnt models utilizing default parameters often exhibit excessive complexity and overfitting. This issue, common in species distribution modeling, hinders accuracy and interpretability because of volatile response curves. Consequently, model optimization is crucial. We optimized the MaxEnt model using the ENMeval package, which systematically minimizes overfitting by evaluating parameter combinations within ecological niches (Gou et al. 2021). Acknowledging that optimal parameters are species‐specific (Kong et al. 2019) and that RM values between 2 and 4 typically yield the best overall performance (Syfert et al. 2013), our optimization identified FC = LQH and RM = 2.5 as the most robust parameters for this study.

*P. nicotianae* exhibits significant regional differences in its suitable habitat under different carbon emission scenarios across various regions of China, reflecting the combined regulatory effects of climate change and geographic environment on the pathogen's ecological niche. In the SSP1‐2.6 low‐carbon emission scenario during 2061–2080, suitable habitats significantly expanded in temperate monsoon climate regions, such as Henan, Shaanxi, Shandong, and Anhui. This change is likely driven by increased winter minimum temperatures, which alleviate the cold stress during the pathogen's overwintering phase (Jia et al. 2024). This climate change promotes the pathogen's recovery from winter dormancy, accelerates the production and release of zoospores, enhances the expression of extracellular enzymes and effector genes, and extends the pathogen's survival period and infection window (Wang et al. 2021). Meanwhile, the relatively gentle topography of the region optimizes soil moisture retention, ensuring a suitable environment for zoospore propagation in the soil, which further expands the suitable habitat range. In the subtropical monsoon humid regions of Yunnan, Guizhou, and Sichuan, stable and abundant precipitation conditions facilitate the completion of the pathogen's life cycle (Romero et al. 2022). The closed topography of the Sichuan Basin enhances humidity retention, maintaining an optimal moisture microenvironment that promotes the germination and active migration of zoosporangia, in line with the pathogen's strong dependence on humid environments. However, in the late period of SSP1‐2.6 (2081–2100), slight reductions in the high‐suitability areas in Yunnan and Sichuan are observed, possibly because of microclimatic seasonal variations in precipitation and moisture heterogeneity caused by topography, which limits the spatiotemporal range of zoospore activity. In the SSP2‐4.5 and SSP3‐7.0 high‐carbon emission scenarios, the warm and humid climate environment likely activates a diverse expression of extracellular effectors, especially in the central Sichuan Basin during 2041–2060, where the pathogen's infection success rate reaches its peak. Under the extreme emission scenario SSP5‐8.5, the suitable habitat for the pathogen exhibits nonlinear dynamics. Additionally, the complex topography of different regions enhances the heterogeneity of the water‐thermal microenvironment, further increasing the spatiotemporal fragmentation of the suitable habitats, reflecting the deep coupling of the pathogen's molecular adaptation mechanisms with environmental regulation. In conclusion, the spatiotemporal changes in the suitable habitats for *P. nicotianae* demonstrate the precise response of the molecular infection mechanism to climatic temperature and humidity changes, while also being influenced by topography's regulation of moisture environments. Future disease prevention and control should be based on multi‐scale coupling between molecular ecology and regional climate geography, combined with dynamic climate data to achieve precise early warning and differentiated management.

In this study, both the MaxEnt and XGBoost‐SHAP models reached the same conclusion, identifying key environmental factors influencing the potential geographic distribution of tobacco, including bio2, bio4, bio6, bio13, bio14, elevation, slope, and land use. Critical factors such as temperature, precipitation, land use, and slope demonstrated a significant impact. For instance, temperature variables exhibited a clear threshold effect, with a marked positive contribution beyond certain critical points, which aligns with the ecological principle that temperature controls physiological processes in species (Aguilar and Lado 2012). Moreover, the results reveal a significant synergistic effect between variables such as slope and precipitation, which together influence soil moisture, reflecting the multi‐factor coupling within the ecosystem (Liu et al. 2023). For terrestrial *Phytophthora* species, temperature and precipitation are fundamental determinants of community diversity and functional composition (Redondo et al. 2018). Multidimensional temperature indicators (e.g., annual mean temperature, temperature difference, minimum temperature of the coldest month). The overwintering ability, infection efficiency, and motile spore processes of tobacco are directly influenced, reflecting a close relationship between the pathogen's physiological adaptation and its ecological distribution (Aguilar and Lado 2012). Warm, humid climates enhance zoospore proliferation and mobility in the water environment, accelerating host infection (Tan et al. 2018). For example, bio2 reflects the daily temperature fluctuation, which is critical for spore germination and initial infection; bio4 and bio6 represent annual temperature variation and extreme winter low temperatures, which limit pathogen distribution in high‐latitude or high‐altitude regions. This aligns with the findings of Zhang et al. (2024), which identified the minimum temperature of the coldest month as a key factor determining the distribution of the medicinal plant Angelica dahurica. Moreover, *Phytophthora* species, being typical warm‐loving organisms, are highly sensitive to extreme temperatures, with higher temperature variability leading to lower suitability. Topographic factors, including elevation and slope, also exhibit a high contribution in the model. High‐altitude regions, with low temperatures and high radiation, are generally unfavorable for *Phytophthora* survival, whereas slope influences soil moisture accumulation and surface runoff, indirectly regulating microenvironmental humidity, thus affecting pathogen colonization and spread. In conclusion, the potential distribution of *P. nicotianae* is influenced by the synergistic effects of multiple climate and topographic variables. Temperature variables determine its overwintering and infection capabilities, whereas precipitation variables regulate the timing of outbreaks. Topographic factors further shape its ecological niche by regulating microenvironmental conditions. These results not only help predict the potential regions affected by tobacco black shank disease but also provide theoretical support for future disease prevention under climate change.

## 5 Conclusions

Within the scope of China, the MaxEnt model combined with the XGBoost‐SHAP model was used to predict the future suitable habitats for *P. nicotianae* . The following key conclusions are drawn.

Through the integrated evaluation of contribution rate, Jackknife analysis, Pearson correlation analysis, and collinearity tests for the 32 candidate variables, the core modeling factors were determined to be bio6, bio14, land use type, bio2, bio4, bio13, and elevation. Among these, bio6 ranks first in both contribution rate and SHAP interpretability, indicating that its sensitivity to extremely low temperatures constitutes the primary constraint on the ecological niche boundary of *P. nicotianae* .

Through testing different feature combinations and regularization parameters (RM), the combination of LQH and RM = 2.5 was identified as the optimal model, effectively balancing model complexity, generalization ability, and discriminative performance. The optimal model achieved an AUC of 0.959 under current climate conditions, and maintained stability in the range of 0.941–0.945 across future SSP climate scenarios, demonstrating strong predictive stability and generalization capability.

Single‐variable response curves and SHAP analysis revealed that the ecological niche of *P. nicotianae* exhibits significant nonlinear responses and ecological thresholds to variables such as temperature, moisture, and topography. Suitability increases rapidly within specific ranges, such as bio6 (temperature near 0°C), elevation around 800–1500 m, bio13 between 150 and 270 mm, and bio14 between 5 and 30 mm, indicating that the population has a limited response to winter low temperatures, extreme moisture, and moderate topographic conditions. Additionally, slope and land use type demonstrate synergistic interaction effects with the main variables, suggesting their role in regulating microenvironments and ecological suitability.

Under future climate change scenarios, the suitable habitat area shows an overall expansion trend. Centroid analysis indicates that the future distribution center will shift toward eastern and northern China. When overlaid with tobacco cultivation areas, the high‐risk areas that require focused prevention and control account for 28.35% of the suitable habitat area for *P. nicotianae* , primarily concentrated in key tobacco‐producing regions such as Yunnan, Guizhou, Henan, and Shandong. This highlights that the actual disease exposure risk is influenced by the crop planting distribution and human activity regulation.

## Author Contributions

**Chaolong Bi:** writing – original draft (equal), writing – review and editing (equal). **Minggang Li:** methodology (equal), software (equal). **Hua Chen:** resources (equal), validation (equal). **Jiangyuan Zhao:** data curation (equal), validation (equal). **Hongbo Zha:** investigation (equal), visualization (equal). **Dexi Wu:** supervision (equal), validation (equal). **Fang Zhao:** conceptualization (equal), data curation (equal). **Peiwen Yang:** conceptualization (equal), project administration (equal).

## Conflicts of Interest

The authors declare no conflicts of interest.

## Supporting information


**Appendix S1:** ece372487‐sup‐0001‐Appendix.pdf.

## Data Availability

The data supporting the results are available in a public repository at: https://doi.org/10.5281/zenodo.15702321.
