# Supplementary material for: The Potential Distribution of Phytophthora nicotianae in China on the Basis of MaxEnt Model Analysis
Source: Ecol Evol. 2025 Dec 3;15(12):e72487. doi: 10.1002/ece3.72487 (PMC12675825; doi:10.1002/ece3.72487)
Supplement: Supplementary file 1 — Appendix S1: ece372487‐sup‐0001‐Appendix.pdf. [file ECE3-15-e72487-s001.pdf]

Appendix to “The Potential Distribution of Phytophthora nicotianae in China Based on MaxEnt Model Analysis”

APPENDIX TABLE 1 | WorldClim-derived bioclimatic variables (Bio 1–Bio 19) employed in this study.

Code	Environmental variable	Unit
Bio 1	Annual Mean Temperature	°C
Bio 2	Mean Diurnal Range (mean of monthly max – min)	°C
Bio 3	Isothermality (Bio 2 / Bio 7 × 100)	%
Bio 4	Temperature Seasonality (standard deviation × 100)	°C
Bio 5	Max Temperature of Warmest Month	°C
Bio 6	Min Temperature of Coldest Month	°C
Bio 7	Temperature Annual Range (Bio 5 –Bio 6)	°C
Bio 8	Mean Temperature of Wettest Quarter	°C
Bio 9	Mean Temperature of Driest Quarter	°C
Bio 10	Mean Temperature of Warmest Quarter	°C
Bio 11	Mean Temperature of Coldest Quarter	°C
Bio 12	Annual Precipitation	mm
Bio 13	Precipitation of Wettest Month	mm
Bio 14	Precipitation of Driest Month	mm
Bio 15	Precipitation Seasonality (coefficient of variation)	—
Bio 16	Precipitation of Wettest Quarter	mm
Bio 17	Precipitation of Driest Quarter	mm
Bio 18	Precipitation of Warmest Quarter	mm
Bio 19	Precipitation of Coldest Quarter	mm

APPENDIX TABLE 2 | Soil environmental factor index.

abbreviation	full name
pH	Soil pH
SOM	Soil Organic Matter
CEC	Cation Exchange Capacity
R	Root Abundance
TN	Total Nitrogen
TP	Total Phosphorus
TK	Total Potassium
AN	Available Nitrogen
AP	Available Phosphorus

AK	Available Potassium
H	Exchangeable Hydrogen
AL	Exchangeable Aluminum
Ca	Exchangeable Calcium
Mg	Exchangeable Magnesium
K	Exchangeable Potassium
Na	Exchangeable Sodium
Dh	Horizon Thickness
LDEP	Profile Depth
SI	Silt Content
CL	Clay Content
GRAV	Gravel Fragments
BD	Bulk Density
POR	Porosity
RC	Rock Content
C1	Consistence
SC	Soil color
Dc	Depth Class or Consistence
PDEP	Pedon Depth
RS	Rock Structure
SW1	Structure Wet or Soil Wetness
Wc	Water Content
Wh	Soil Color Dry

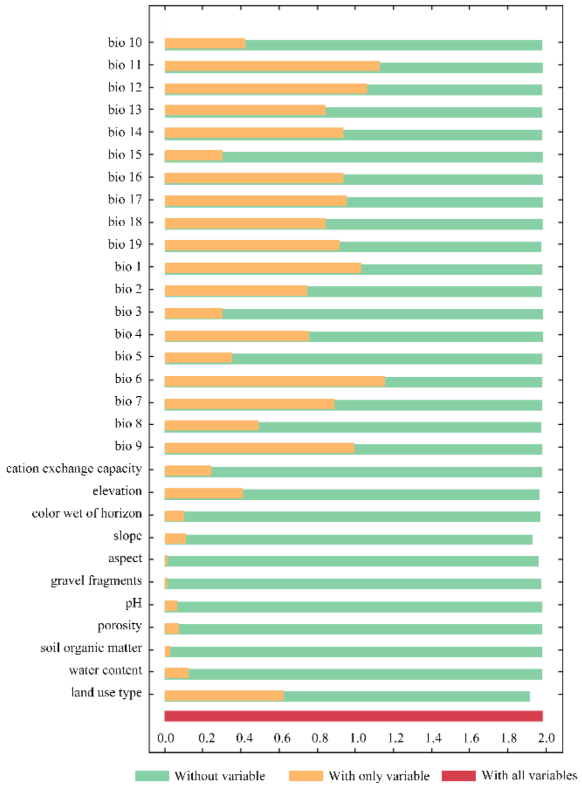

APPENDIX FIGURE 1 | Regularized training-gain curve.

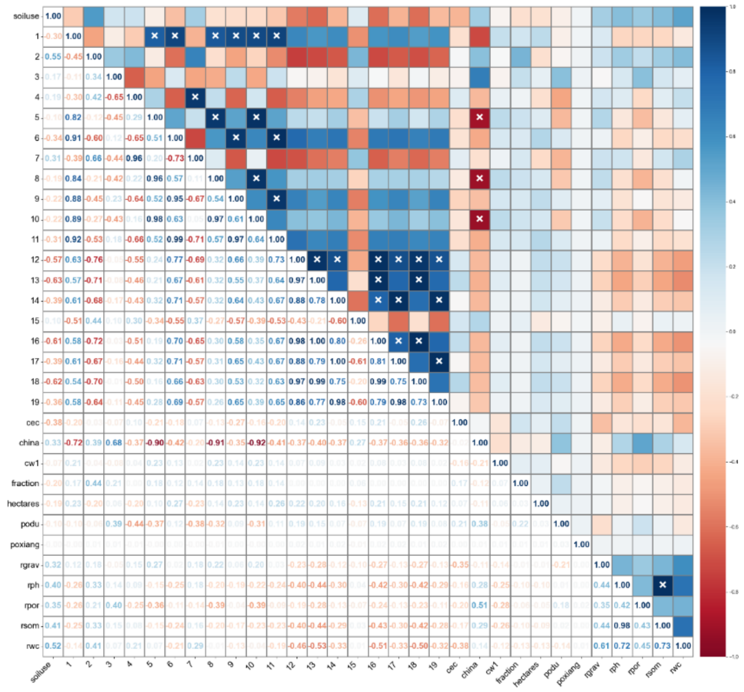

APPENDIX FIGURE 2 | Correlation screening results of environmental factors.

Note: The figure shows that "×" represents a correlation absolute value greater than 0.8.

APPENDIX TABLE 3 | VIF Analysis Results of Environmental Factors

Variables	VIF	Variables	VIF
Bio 1	1.63E+03	Bio 7	6.48E+13
Bio 10	4.51E+03	Bio 8	3.97E+01
Bio 11	1.24E+04	Bio 9	3.54E+01
Bio 12	1.63E+02	cec	2.11E+00
Bio 13	1.14E+02	elevation	4.21E+01
Bio 14	9.62E+01	cw	1.31E+00
Bio 15	4.54E+00	fraction	3.57E+02
Bio 16	2.66E+02	hectares	3.58E+02
Bio 17	1.29E+02	slope	1.66E+00
Bio 18	9.60E+01	aspect	1.01E+00
Bio 19	4.46E+01	grav	1.96E+00
Bio 2	9.15E+01	pH	4.13E+01
Bio 3	2.50E+01	por	2.34E+00
Bio 4	5.41E+03	som	4.31E+01
Bio 5	3.35E+13	wc	3.96E+00
Bio 6	8.34E+13	land use type	2.03E+00

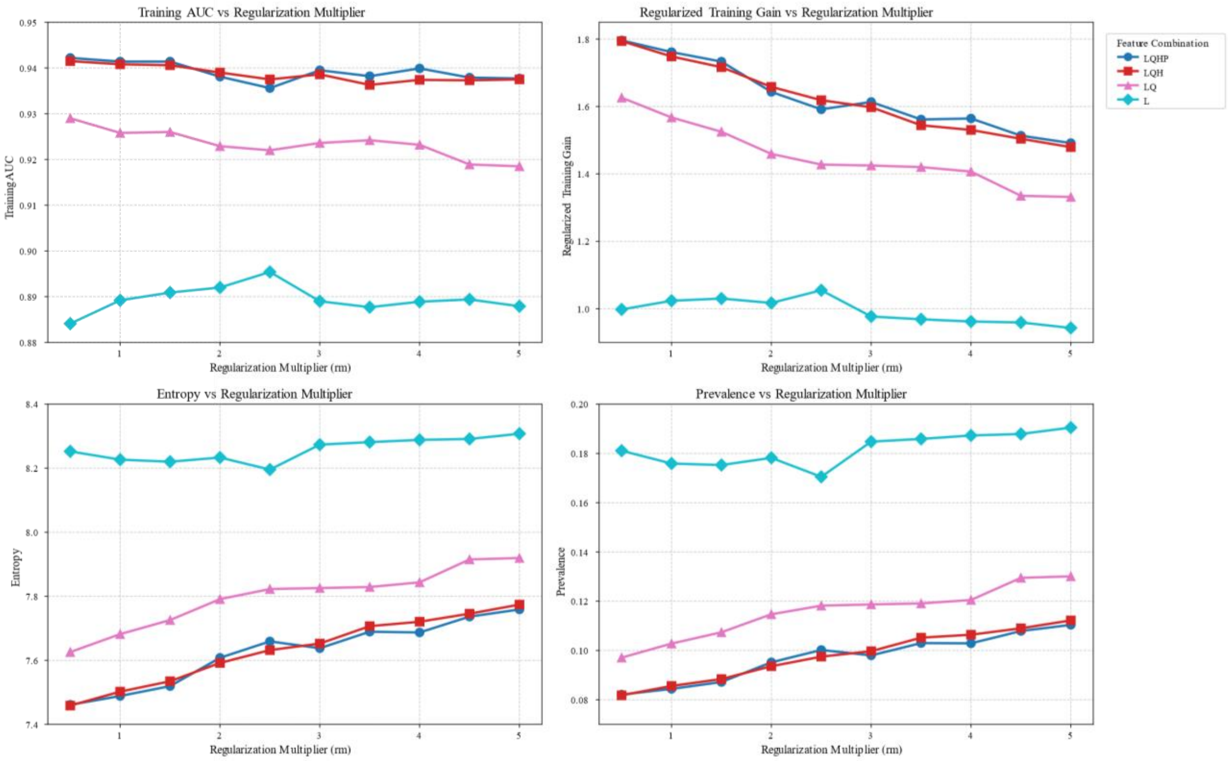

APPENDIX FIGURE 3 | MaxEnt tuning results across regularization multipliers and feature sets.

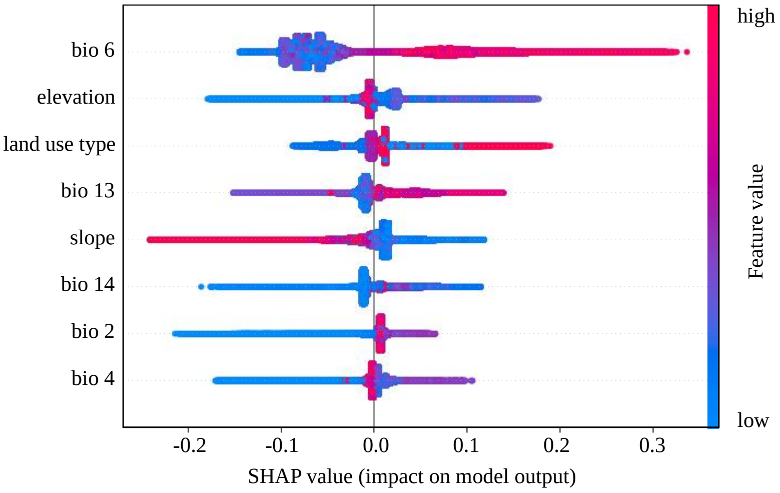

APPENDIX FIGURE 4 | SHAP summary plot of environmental variables**.**

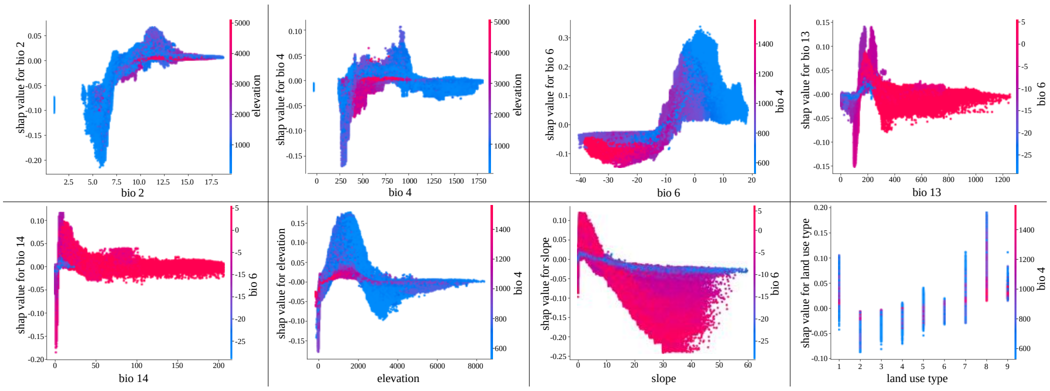

APPENDIX FIGURE 5 | SHAP dependence plots of environmental variables.

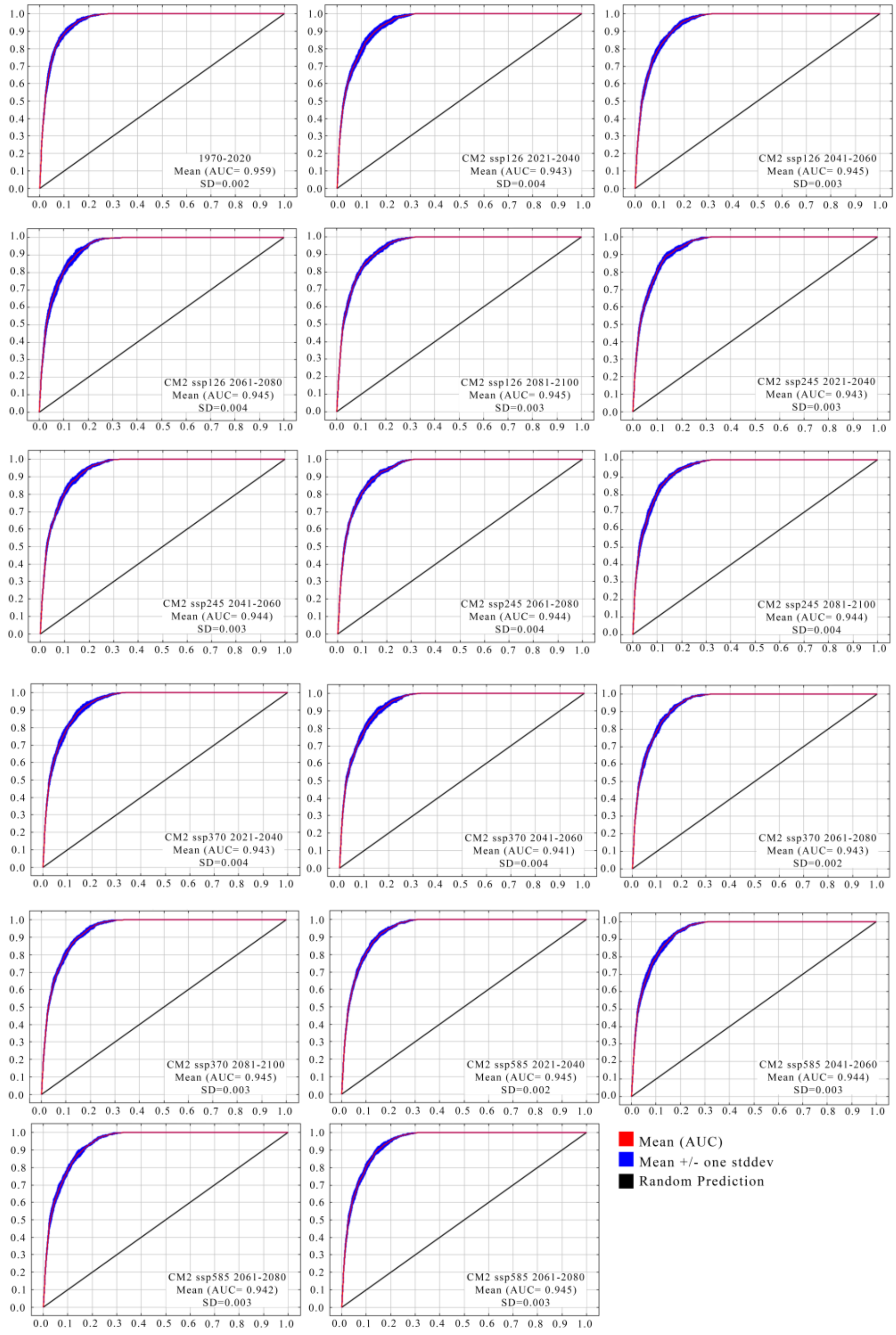

APPENDIX FIGURE 6 | ROC curves of MaxEnt model predictions. Note: The y-axis represents sensitivity, and the x-axis represents 1 − specificity (Fractional Predicted Area).

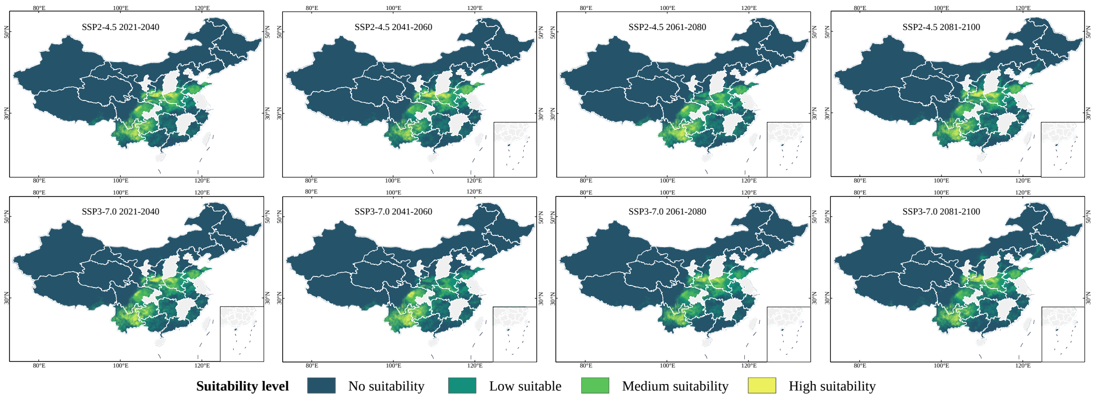

APPENDIX FIGURE 7 | Potential geographical distribution of *P. nicotianae* under current climatic conditions.

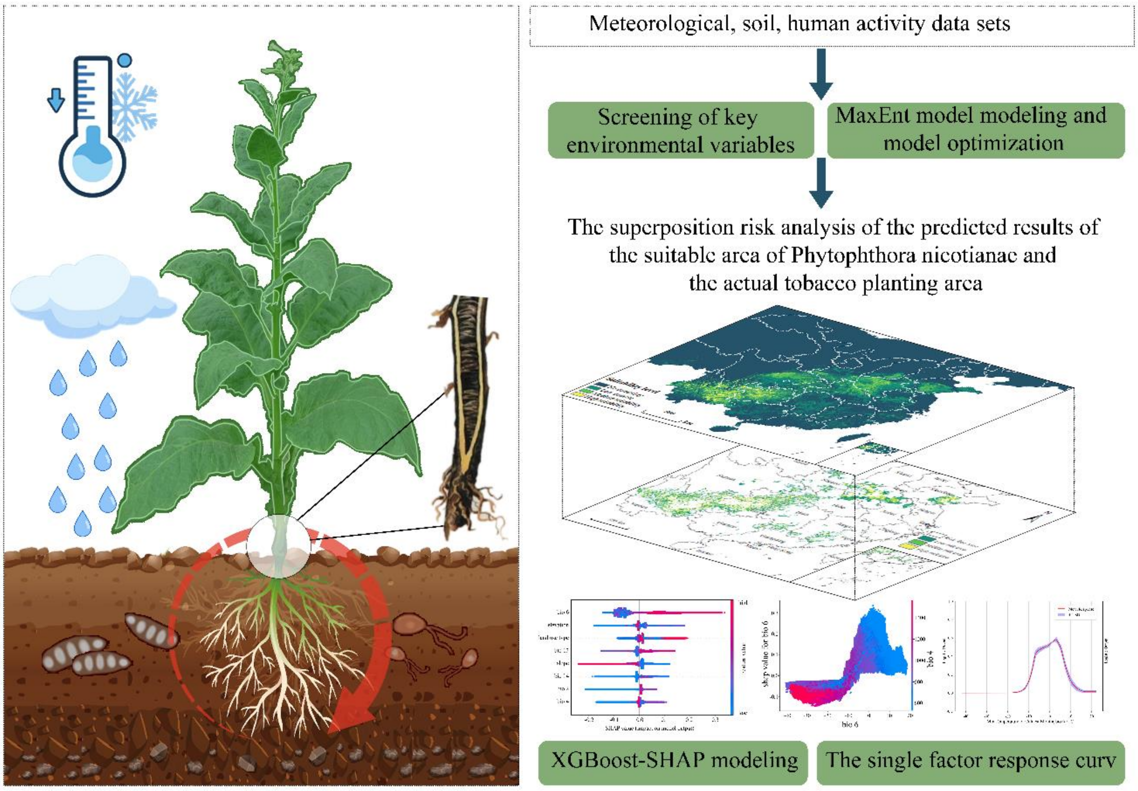

APPENDIX FIGURE 8 | Graphical Abstract.
